# Susceptibility to ozone-induced airway inflammation is associated with decreased levels of surfactant protein D

**DOI:** 10.1186/1465-9921-7-85

**Published:** 2006-06-01

**Authors:** S Kierstein, FR Poulain, Y Cao, M Grous, R Mathias, G Kierstein, MF Beers, M Salmon, RA Panettieri, A Haczku

**Affiliations:** 1 University of Pennsylvania, Philadelphia, PA, USA; 2 University of California, Davis, CA, USA; 3 GSK, King of Prussia, PA, USA

## Abstract

**Background:**

Ozone (O_3_), a common air pollutant, induces exacerbation of asthma and chronic obstructive pulmonary disease. Pulmonary surfactant protein (SP)-D modulates immune and inflammatory responses in the lung. We have shown previously that SP-D plays a protective role in a mouse model of allergic airway inflammation. Here we studied the role and regulation of SP-D in O_3_-induced inflammatory changes in the lung.

**Methods:**

To evaluate the effects of O_3 _exposure in mouse strains with genetically different expression levels of SP-D we exposed Balb/c, C57BL/6 and SP-D knockout mice to O_3 _or air. BAL cellular and cytokine content and SP-D levels were evaluated and compared between the different strains. The kinetics of SP-D production and inflammatory parameters were studied at 0, 2, 6, 12, 24, 48, and 72 hrs after O_3 _exposure. The effect of IL-6, an O_3_-inducible cytokine, on the expression of SP-D was investigated *in vitro *using a primary alveolar type II cell culture.

**Results:**

Ozone-exposed Balb/c mice demonstrated significantly enhanced acute inflammatory changes including recruitment of inflammatory cells and release of KC and IL-12p70 when compared with age- and sex-matched C57BL/6 mice. On the other hand, C57BL/6 mice had significantly higher levels of SP-D and released more IL-10 and IL-6. Increase in SP-D production coincided with the resolution of inflammatory changes. Mice deficient in SP-D had significantly higher numbers of inflammatory cells when compared to controls supporting the notion that SP-D has an anti-inflammatory function in our model of O_3 _exposure. IL-6, which was highly up-regulated in O_3 _exposed mice, was capable of inducing the expression of SP-D *in vitro *in a dose dependent manner.

**Conclusion:**

Our data suggest that IL-6 contributes to the up-regulation of SP-D after acute O_3 _exposure and elevation of SP-D in the lung is associated with the resolution of inflammation. Absence or low levels of SP-D predispose to enhanced inflammatory changes following acute oxidative stress.

## Background

Ozone (O_3_), an ubiquitous, oxidizing, and highly toxic air pollutant is generated photo-chemically from nitric oxides and hydrocarbons. O_3 _is associated with an immediate impairment of lung function and contributes to increased morbidity in patients with asthma and chronic obstructive pulmonary disease (COPD) [1,2]. Even in healthy subjects, short-term exposure to O_3 _increases levels of the vascular adhesion molecules P-selectin and ICAM-1 in airway lavages and bronchial tissue and induces influx of neutrophils and mast cells [3]. In mouse, it has been shown that the quality and time course of the cellular response vary considerably between different inbred strains. Some strains like 129/J and DBA/2J respond with an early peak of polymorphonuclear cells six hours after exposure, whereas C57BL/6J mice reach the peak of inflammation 24 hrs after exposure. Additionally, A/J and C3H/HeJ mice respond with only minimal cellular influx [4]. The O_3_-induced acute pathological changes are characterized by an influx of neutrophils and airway hyperresponsiveness (AHR). Long-term or chronic exposure to O_3_, however, attenuates inflammatory responses, a phenomenon referred to as adaptation [5]. The early adaptive response (within 18 hrs after O_3 _exposure) is largely IL-6 dependent but the late adaptive response (several days after exposure) involves mobilization of pulmonary antioxidants and leads to hypertrophy and metaplasia of epithelial cells in the upper as well as in the lower respiratory tract [5-8]. The mechanisms influencing the severity of the O3-induced pulmonary reaction and the molecules involved in the modulation of this response are yet to be fully determined.

Surfactant protein-D (SP-D), a pattern-recognition molecule of the pulmonary innate immune system, enhances the phagocytosis and clearance of various inhaled pathogens, allergens, and apoptotic cells in the lung and serves as a potent immuno-modulator [9-11]. SP-D possesses anti- as well as pro-inflammatory functions depending on binding specificities and orientation against cell surface receptors [12]. SP-D also inhibits T-cell activation and allergic inflammatory events and it may function as a local regulator of a T-helper type 2 (Th2) inflammation [13-15]. The expression of SP-D is regulated developmentally but SP-D levels increase from baseline constitutive expression under a variety of lung inflammatory conditions [16,17]. We have previously shown that SP-D production induced during allergic inflammation is mediated by the Th2 cytokine IL-4 [13,18]. However, little is known regarding the role and regulation of SP-D in non-antigen-related inflammatory changes of the lung. Recently, Casey and colleagues proposed an anti-inflammatory role of SP-D in a mouse model of bleomycin-induced lung injury [19]. Since different mouse strains not only vary in their airway responses to O_3 _but also express different levels of SP-D, we hypothesized that there is a causal relationship between these two characteristics. To test our hypothesis and to better characterize the role SP-D may play in O_3_-induced inflammation, we used mice with reportedly different SP-D levels [13] and mice lacking SP-D [20] We found that mice expressing high levels of SP-D had significantly less severe inflammatory responses as compared to mice with low or no SP-D. Additionally, the O_3_-inducible cytokine IL-6 selectively induced the expression of SP-D *in vitro*.

## Methods

### Animals

All experimental animals used in this study were housed under pathogen-free conditions. Experiments were performed between 8 and 12 weeks of age. Animals received water and food *ad libitum*. The protocols were approved by the Institutional Animal Care and Use Committee of the University of Pennsylvania and GlaxoSmithKline.

### Modes of O_3 _exposure

To evaluate the effects of O_3 _exposure in mouse strains with different SP-D levels, we used Balb/c and C57BL/6 mice and exposed them to 3.0 ppm O_3 _for a 2 hrs period. BAL SP-D levels, cellular and cytokine content were evaluated 6 hrs later. To define the kinetics of the O_3_-induced inflammation and SP-D production in more detail, C57BL/6 mice (Jackson Laboratory, Bar Harbor, ME) were exposed to 3.0 ppm O_3 _or air for a 2 hrs period and studied 2, 6, 12, 24, 48, and 72 hrs later. Finally, to study the effects of a complete lack of SP-D, SP-D knockout mice [20] were exposed to either 3.0 ppm O_3 _for 2 hrs or to 0.5 ppm O_3 _for 24 hrs. BAL was performed 12 hrs (2 hrs exposure) and 24 and 48 hrs (24 hrs exposure) later. In all experiments age- and strain-matched controls were exposed to room air concurrently. The levels and exposure times were based on a previous pilot study (unpublished) and were chosen to accommodate all three different mouse strains and to allow us to study and compare the temporal inflammatory changes. After exposure, groups of mice (n = 6) were euthanized and BAL was performed.

### Bronchoalveolar lavage (BAL)

#### Differential cell count

BAL was performed as previously described [13]. Briefly, mice were euthanized with an i.p. injection of a mixture of ketamine and xylazine (100 mg/kg and 20 mg/kg respectively). A tracheotomy was performed and the trachea was canulated with a 20 gauge blunt end needle. Lavage was carried out once with 0.7 ml and twice with 1 ml sterile PBS. The recovered BAL from three lavages was pooled. BAL was centrifuged at 4°C for 10 min. at 400 *g *and the pellet was resuspended in 1 ml of PBS. Total cell counts were determined from an aliquot of the cell suspension. Differential cell counts were done on cytocentrifuge preparations (Cytospin 3; Thermo Shandon, Pittsburgh, PA) stained with Kwik™Diff (Thermo Shadon, Pittsburgh, PA), and 200 – 500 cells were counted from each individual.

#### Cytokine assays, SP-D Western blots and ELISA

Cytokine and chemokine levels in the cell-free BAL were determined as part of a Luminex^®^100™ assay System (Luminex Corporation, Austin, TX) and Endogen^®^SearchLight™ Mouse Cytokine and Chemokine arrays (Pierce Biotechnology Inc., IL), respectively, and was performed according to the manufacturer's instructions.

Total protein from cell free supernatant of the BAL fluids was assessed using the Bradford Assay (BioRad, CA). Western blots for SP-D levels in cell-free BAL fluid were performed as previously described [18]. Briefly, 4 μg of total protein were loaded and run on an SDS-PAGE and transferred onto nitrocellulose membranes. Membranes were incubated with a rabbit polyclonal anti-SP-D antibody (Chemicon Int., Temecula, CA), followed by incubation with horseradish peroxidase conjugated goat anti-rabbit IgG (Bio-Rad, CA). Specific binding was visualized by enhanced chemiluminescence with ECL Kit (Amersham, IL). The intensity of the signals was quantified with GelPro Analyzer 4.0 (Media Cybernetics Inc., NJ) software. The band density values obtained from individuals were expressed as percentage of the band intensities of treated animal to non-treated, naïve samples. To be able to compare different mouse strains the mean baseline levels in each strain were assigned the value 100 % (± SEM).

SP-D protein recovered from BAL was quantified by ELISA using an in-house rabbit polyclonal anti-SP-D antibody [18]. Aliquots of the BAL samples neat or diluted with blocking buffer (1 % BSA, 2 % normal goat serum, 0.5 % Tween-20 in Dulbecco's Phosphate-buffered saline) were applied to 96-well Nunc-Immuno Max iSorp plates (Nalgene Nunc International, Denmark). Each assay plate included a standard of purified SP-D peptide (0.31 to 40 ng/ml) [18]. Polyclonal anti-SP-D antiserum was applied as a primary antibody (1:10,000) and horseradish peroxidase conjugated goat anti-rabbit IgG (1:1,000) was used as the secondary antibody. Colorimetric detection was performed using ABC reagent (Vectastain ABC kit, Vector Laboratories, Burlingame, CA) according to the manufacturer's instructions. Color intensity was measured at 405 nm using an automated microplate reader (Bio-Rad, Hercules, CA) and analyzed with Bio-Rad Microplate manager software. Overlapping serial dilution curves of the SP-D peptides and the purified SP-D protein showed a semi-logarithmic relationship between OD and concentration. ELISA for SP-A was performed as published previously [13].

#### Alveolar type II cell culture

Lung alveolar type II cells were isolated from neonatal Sprague-Dawley rats (Charles River Laboratories, Wilmington, MA) as previously described [3,18]. Our method yields approximately 60% of type II cells (positive for the lamellar body protein ABCA3). Major contaminating cell types are macrophages and fibroblasts. The viability of type II cells in our culture system is about 85–95 %. Cells were cultured in serum-free Weymouth's MB 752/1 medium (Invitrogen, Carlsbad, CA) containing DCI [Dexamethasone (10 nM), 8-Br-cAMP (100 μM) and Isobutylmethylxantine (100 μM) all from Sigma, St. Luis, MO)] in the presence or absence of IL-6 (BD Pharmingen, San Diego, CA) for 4 days. Western blots for intra-cellular SP-D were performed as described above.

### Data analysis

Statistical analysis was performed with Prism4 software (GraphPad Inc., San Diego, CA). Multiple comparisons were performed by one-way-ANOVA followed by Barlett's test or Post test for linear trend. Student *t*-test was used for two-group comparisons. Data are expressed as mean ± SEM, p < 0.05 was considered statistically significant.

## Results

### A relative SP-D deficiency in Balb/c mice was associated with exaggerated inflammatory changes 6 hrs after O_3 _exposure

**Figure 1:**
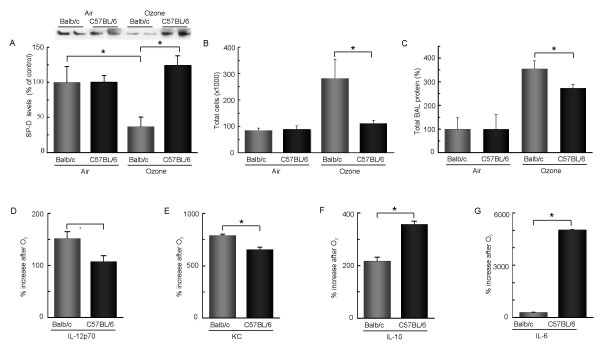
**A decrease in BAL SP-D levels was associated with significantly increased inflammation in Balb/c mice 6 hrs after O3 exposure**. Groups of Balb/c (grey bars) and C57BL/6 (black bars) mice were exposed to O_3 _or room air for 2 hrs and BAL was performed 6 hrs later. **(A) **Ozone exposed Balb/c mice had significantly reduced SP-D levels as compared to air exposed Balb/c or O_3 _exposed C57BL/6 mice. SP-D was detected by Western blot analysis of the cell-free supernatant of the BAL (top panel) and was performed using our in-house rabbit polyclonal anti-SP-D antibody. Two representative samples out of a total of six are shown in each group. SP-D expression was quantified by densitometric analysis. Results are expressed as % of naïve control levels. * p = 0.0249 *vs*. room air; p = 0.0027 *vs*. C57BL/6. **(B) **Balb/c mice had significantly higher numbers of inflammatory cells in their BAL as compared to C57BL/6 mice. Cells were counted using a Coulter counter and results are expressed as cell number/ml (*p = 0.0316). **(C): **The BAL total protein content was significantly higher in Balb/c mice compared to C57BL/6 mice. Total protein was measured by Bradford assay in the cell-free supernatant *p = 0.028. Absolute protein contents were 919,6 (± 51,4) and 3262,3 (± 281.0) in air and O_3 _exposed Balb/c mice, respectively, and 772 (± 26.4) and 2110 (± 36.4) in air and O_3 _exposed C57BL/6 mice, respectively.**(D-G): **Cytokine expression was studied by Endogen^®^SearchLight™ and Luminex^®^100™ technologies. O_3 _induced the release of IL-12p70 (34 pg/ml ± 4 in Balb/c, 18.7 pg/ml ± 1 in C57BL/6), IL-6 (2,393 pg/ml ± 119 in Balb/c, 412 pg/ml ± 68.7 in C57BL/6), IL-10 (110 pg/ml ± 16 in Balb/c, 14.2 pg/ml ± 1.6 in C57BL/6) and KC (1,896 pg/ml ± 224 in Balb/c, 136.8 pg/ml ± 27.7 in C57BL/6). Cytokine and chemokine levels are expressed as % increase from control levels. The O_3_- induced pro-inflammatory cytokine IL-12p70 and KC levels were significantly higher (*p = 0.0134 and *p = 0.0001, respectively) whereas the immunosuppressive IL-10 and the immunoregulatory IL-6 levels were significantly lower (*p < 0.0001 and *p < 0.0001, respectively) in Balb/c mice than in C57BL/6 mice. **(A-G): **Mean ± SEM of n = 6 in each groups.

We have previously reported that SP-D levels in Balb/c and C57BL/6 mice differ under normal conditions as well as upon allergen sensitization and challenge [13]. Since SP-D is a potent immuno-regulator we were interested in evaluating whether these mouse strains would show quantitative differences in their inflammatory response to O_3_. In these experiments BAL SP-D levels in the different moue strains were normalized to 100%, i.e. their mean baseline level. We previously published results of a direct comparison between naïve Balb/c and naïve C57BL/6 mice in which Western blot analysis demonstrated that C57BL/6 mice had approximately twice as much SP-D as Balb/c mice [13]. As shown in Fig. 1A, O_3_-exposure caused a significant drop in SP-D levels in Balb/c (but not in C57BL/6) in comparison with air exposed controls (p = 0.0249). Six hours after O_3 _exposure, the amount of SP-D recovered from the BAL (and normalized to the baseline) was significantly lower in Balb/c mice compared with C57BL/6 mice (p = 0.0027).

Balb/c mice also had significantly more inflammatory cells (mainly neutrophils, approx. 50 % of total cell counts) compared to C57BL/6 mice (p = 0.0316; Fig. 1B). Moreover, Balb/c mice had significantly higher total protein content in their BAL as compared to C57BL/6, indicating more severe lung injury (p = 0.028; Fig. 1C). The levels of the pro-inflammatory cytokine IL-12p70 and the neutrohpil chemo-attractant KC were significantly higher in Balb/c as compared to C57BL/6 mice (p = 0.0134 and p = 0.0001, respectively; Fig. 1D–E) after O_3 _challenge. In contrast, C57BL/6 mice released more IL-10 and IL-6 (p < 0.0001 and p < 0.0001, respectively; Fig. 1F–G). Absolute cytokine levels are indicated in the figure legend.

### Kinetics of SP-D during O_3_-induced inflammatory changes

**Figure 2:**
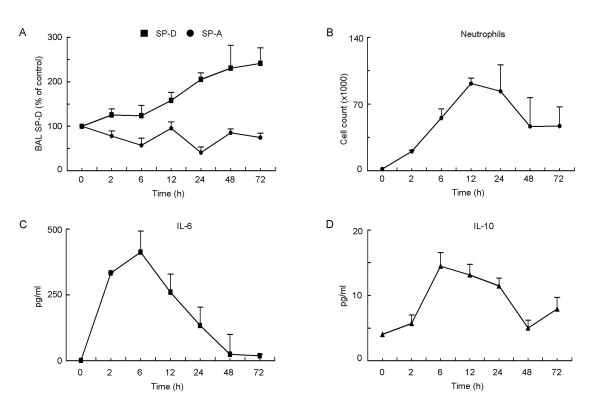
**Kinetics of O_3_-induced SP-D and inflammatory changes in C57BL/6 mice**. Groups of C57BL/6 mice were exposed to 3 ppm O_3 _or room air and studied 0, 2, 6, 12, 24, 48, and 72 hrs later. **(A): **SP-D and SP-A recovered from the BAL were quantified by an in-house ELISA. Results are expressed as % change from naïve controls. SP-D levels gradually increased until the 72 hrs time point (p = 0.0022, ANOVA and post test for linear trend) whereas SP-A levels did not change significantly. **(B)**: The number of neutrophilic granulocytes was assessed by counting total number of BAL cells in the BAL fluid and performing differential cell counting on Kwick™Diff cytospin preparations. Results are expressed as absolute cell number/ml of BAL fluid. Neutrophilic inflammation peaked around 12 hrs post exposure (p < 0.0001) and largely resolved by 72 hrs after O_3 _exposure. **(C-D) **IL-6 and IL-10 levels were assessed as part of a Luminex^®^100™ assay and showed significant increases 2–6 hrs after O_3 _exposure (p < 0.0001) and 6–24 hrs after O_3 _exposure (p = 0.0007), respectively. **(A-D): **Mean ± SEM of n = 6 in each groups.

To study the kinetics of SP-D changes in the context of O_3_-induced inflammation we used C57BL/6 mice (the "SP-D high" strain) and followed the onset and resolution of the inflammation at 0, 2, 6, 12, 24, 48, and 72 hrs after O_3 _exposure. ELISA for SP-D and SP-A recovered from the BAL fluid of O_3_-exposed mice showed significant elevation of SP-D levels with approximately 50 % increase from baseline 12 hrs post-exposure. SP-D continued to increase until the last time point of the experiment (72 hrs) when SP-D levels were about 150 % above control levels (p = 0.0022; Fig. 2A). SP-A levels on the other hand did not change significantly. The SP-D ELISA results were verified using Western blot analysis. The two different methods showed a significant positive Spearman correlation r = 0.86 (p = 0.0238). Inflammatory cells were detected 2 hrs after O_3_-exposure, and the numbers were significantly increased compared to naïve animals and peaked around 12 hrs post-exposure (p < 0.0001; Fig. 2B). A slight increase in the numbers of lymphocytes (up to 3 % of total cell counts) was also observed with a time course comparable with that of neutrophilic cells (p = 0.0003; not shown). Eosinophil counts showed a transient peak at 6 hrs, but their number remained less than 1 % of total cell counts at all time points (not significant; not shown). Airway neutrophilia resolved markedly within 72 hrs after O_3 _exposure indicating an inverse relationship between the rise of SP-D levels and the decrease of inflammatory cells, including neutrophils and lymphocytes (Fig. 2A and 2B). There was however no statistical correlation between these parameters. The neutrophil influx was preceded by a significant increase in KC levels but there was no significant correlation between this chemokine and neutrophil recruitment. Release of KC into the airways started 2 hrs after O_3 _challenge and reached a peak at 6 hrs (p < 0.0001; not shown). KC levels were back to baseline by 24 hrs. Interestingly, IL-6 was highly induced, with a peak at the 6 hrs time point and a return to baseline levels 48 hrs post-exposure (p < 0.0001; Fig. 2C). Release of the anti-inflammatory cytokine IL-10 was delayed by several hours. IL-10 levels were slightly but significantly increased with highest values between 6–24 hrs after O_3 _challenge (p = 0.0007; Fig. 2D).

### SP-D deficient mice have increased cellular inflammation following O_3 _exposure

**Figure 3:**
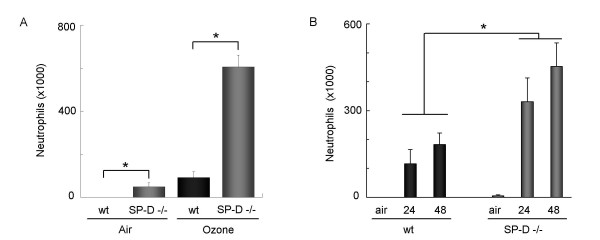
**SP-D deficient mice have increased airway inflammation following O_3 _exposure**. (A) SP-D -/- mice and age-matched C57BL/6 controls were exposed to 3 ppm O_3 _for 2 hrs or (B) to 0.5 ppm for 24 hrs. Influx of neutrophilic granulocytes was assessed on cytospin preparations stained with Kwick™Diff. In both models cellular inflammation in SP-D -/- mice was significantly higher compared to wt mice (A) Student *t*-test *p = 0.0106 (B) ANOVA and Barlett's test *p = 0.0082.

To further evaluate the anti-inflammatory role of SP-D in the O_3_-induced immune response we used SP-D deficient mice and compared them with age- and sex-matched C57BL/6 wild-type controls. SP-D deficient mice showed a baseline inflammation that was further increased after O_3 _exposure. The O_3_-induced cellular response was significantly higher in SP-D -/- mice compared to wt C57BL/6 mice 12hrs after acute O_3 _exposure (p = 0.0106; Fig. 3A). In addition, when mice were exposed to O_3 _for 24 hrs (0.5ppm O_3_), a sub-acute exposure, this finding was confirmed, since SP-D -/- mice had increased numbers of inflammatory cells both 24 and 48 hrs after cessation of O_3 _exposure (Fig. 3B, p = 0.0082). Unlike after acute O_3 _exposure (Fig. 2B), neither the wild type nor the SP-D-/- mice showed signs of resolution of cellular infiltration at the 48 hrs time point after sub-acute exposure. On the contrary, inflammatory cell numbers were further increased (Fig. 3B).

### IL-6 selectively induces the production of SP-D *in vitro*

**Figure 4:**
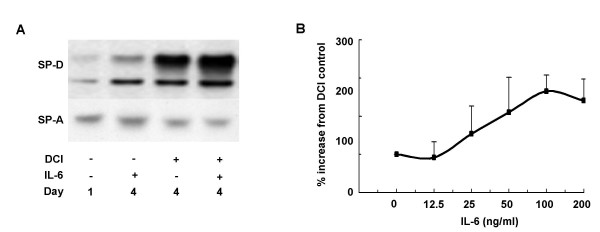
***In vitro *production of SP-D by alveolar type II cells after IL-6 stimulation**. (A) Primary rat alveolar type II cells were cultured in the presence or absence of DCI and IL-6 for up to 4 days and intra-cellular SP-D and SP-A levels were assayed by Western blot analysis. Day 0 SP-D and SP-A signals were obtained from freshly isolated alveolar type II cells. To maintain the SP-D producing phenotype, cells have to be cultured in the presence of DCI (10 nM dexamethason, 100 nM IBMX, 10 nM cAMP) that in turn stimulates SP-D production. IL-6 selectively stimulated the production of SP-D but did not induce up-regulation of SP-A. (B) Increase of SP-D after IL-6 stimulation at day 4 was slightly dose dependent. All levels were compared to non-stimulated alveolar type II cells cultured in DCI containing medium (DCI control).

We have shown previously that SP-D induction in allergic inflammation is dependent on IL-4 and IL-13 [15,18]. Although, none of these Th2 type cytokines was induced in the present model of O_3 _challenge, the production of SP-D was highly up-regulated. Therefore we tested the possibility that one of the O_3_-inducible cytokines is capable of promoting SP-D expression. Since IL-6 showed the most pronounced changes following O_3 _challenge, and since it is a pluripotent immuno-regulatory cytokine, we chose to investigate its effects on SP-D gene expression *in vitro*. As shown in Fig. 4A, *in vitro *stimulation of primary rat alveolar type II cells revealed that IL-6 is indeed capable of directly up-regulating SP-D production. The effect of IL-6 was dose dependent (Fig. 4B) and selective for SP-D, because SP-A production was not changed (Fig. 4A).

## Discussion

Our results confirm the findings of other investigators showing that acute O_3 _exposure induces a rapid onset and resolution of airway inflammatory changes characterized by a KC-driven neutrophilic inflammation and moderately increased numbers of lymphocytes, eosinophils and macrophages [4,6]. Altered levels of surfactant protein D have been reported in association with a number of different pathological conditions of the lung [13,15,18,21-24]. Here we show that O_3 _exposure induces a delayed expression of SP-D. Our data also show that the susceptibility to O_3_-induced inflammatory changes varies between different mouse strains and appears to be associated with different levels of SP-D. C57BL/6 mice that express high levels of SP-D also produce high levels of the anti-inflammatory cytokine IL-10 and high levels of IL-6. In contrast, Balb/c mice release significantly more KC and IL-12p70. Elevated levels of SP-D are associated with the resolution of the O_3_-induced inflammation and low levels or lack of SP-D predispose to a severe inflammatory response.

The drop in SP-D levels seen in Balb/c mice 6 hrs after O_3 _exposure could be due to a direct damage and/or apoptosis of SP-D producing epithelial cells [25]. It is likely that this acute phenomenon affects stored SP-D only, because *de-novo *mRNA expression occurs only about 6 hrs after allergen challenge or pulmonary infection and increased levels of SP-D protein were only detected at about 12 hrs (Fig. 2A) [13,18,26]. Additionally, the size of the extracellular SP-D pool might be important in the protection from O_3_-induced epithelial injury. Although the authors did not specifically investigate the role of SP-D, Li and colleagues demonstrated that endotoxin pre-treatment, which is expected to induce SP-D production, protects against O_3_-induced cell death and pulmonary inflammation [27,28]. This could explain why C57BL/6 mice with their higher levels of SP-D were more protected from the acute effects of O_3_. Although it has been shown previously that different mouse strains vary in their acute O_3_-induced pulmonary response [4], no host factors responsible for the individual susceptibility have been identified. Savov and co-workers identified several chromosomal regions that appeared to be associated with the physiologic and biologic phenotypes [4]. Their *in silico *genome scan indicates that a locus between 30 and 40 megabases (Mb) on mouse chromosome (MMU) 14 contains one or more relevant genes. It is noteworthy, that the gene coding for SP-D is located on MMU 14 in the area of 37.2 Mb. Additionally, two recent reports identified sequence polymorphisms in the human SP-D gene that lead to differences in constitutive serum levels and influence the multimeric assembly and function of SP-D protein [29,30].

Neutrophils play a vital role in the pulmonary host defense. However, due to their release of large amounts of histo-toxic and pro-inflammatory agents, these cells can cause significant tissue damage. Hence, a stringent control of neutrophil priming, recruitment, activation, apoptosis and clearance is crucial to confine tissue damage [31]. Mice genetically deficient in SP-D have chronic inflammation and hyper-activated macrophages further strengthening the important role of SP-D as a local regulator of the innate immune response [20,32,33]. In a recent publication White and colleagues demonstrated that SP-D may either inhibit or enhance neutrophil respiratory burst responses to influenza A virus. Their data also suggest that the effects of SP-D are modulated by the presence of other respiratory innate immune proteins such as SP-A, and on the multimerization state of SP-D [34]. Other studies show that administration of recombinant SP-D enhances the up-take of apoptotic cells and reduces the production of pro-inflammatory cytokines [35,36]. In line with those results, our study shows that levels of KC, the main chemo-attractant for neutrophils, significantly dropped in concert with a significant elevation of SP-D 12 hrs after O_3 _exposure in C57BL/6 mice. However, whether or not SP-D has a direct regulatory effect on KC remains to be determined and is the current focus of our studies.

Ozone exposure does not induce the release of classical Th2 type cytokines such IL-4 or IL-13 which have been shown to stimulate the expression of SP-D [18]. However, in our study we show that O_3 _induced a significant rise in BAL SP-D suggesting that there are other mechanisms to promote SP-D expression during a non-allergen induced inflammation. Indeed, our *in vitro *studies using rat alveolar type II cells show that IL-6 is capable of inducing SP-D production. In a different model of airway inflammation, elicited by allergic sensitization and challenge, we have previously shown that a rapid release of pro-inflammatory cytokines is followed by a relatively slow, gradual elevation of SP-D protein levels in the airways, with a peak 48 hours after allergen challenge [15]. In accordance to that, SP-D protein levels were still increased at 48 and 72 hrs when IL-6 levels were already back to normal. IL-6 can transduce its signal either via C/EBPβ or via Stat3 activation. The SP-D promoter region harbours binding sites for both of these transcription factors [37]. Whether IL-6-dependent SP-D gene expression is promoted by C/EBPβ or Stat3 or synergistically by both of them remains to be clarified. Interestingly, IL-6 deficient mice have significantly less BAL protein, neutrophils and soluble TNF receptors after exposure to sub-acute levels of O_3_[38]. On the other hand mice over-expressing IL-6 are protected from lung injury caused by chronic hypoxia [39]. These findings point out the pluripotent functions of IL-6 as an anti- as well as a pro-inflammatory cytokine. The reports by Johnston *et al*. and Ward *et al*. also provide indirect support of our findings that IL-6 is important in launching a protective pulmonary response. Our results showed that the neutrophil chemoattractant KC and the immunosuppressive cytokine, IL-10 were also elevated in the BAL fluid after O_3 _exposure. While the possibility was raised that these mediators could contribute to the up-regulation of SP-D, we found no evidence that lung epithelial cells would express receptor or show any functional response to exogenous IL-10 or KC [40].

## Conclusion

Strain dependent differences in SP-D production are associated with differences in the severity of the inflammation. The importance of SP-D in the protection against the initial O_3_-induced injury and during the resolution of the inflammation is confirmed in SP-D knockout mice, as the absence of SP-D resulted in enhancement of the inflammation in these animals. We propose that IL-6 may contribute to the up-regulation of SP-D expression, which in turn inhibits pro-inflammatory changes and promotes resolution of the inflammation following O_3 _exposure.

## Competing interests

The author(s) declare that they have no competing interests.

## Authors' contributions

SK participated in the animal experiments, BAL cell counts, Western blots, data analysis and prepared the manuscript.

FRP supervised the animal experiments using SP-D deficient mice and advised on data analysis.

YC participated in most of the animal experiments and developed the ELISA for SP-D.

MG performed the time course study in C57BL/6 mice.

RM performed the animals experiments using SP-D -/- mice.

GK developed a template for statistical data analysis and participated in the preparation of the manuscript.

MFB gave helpful advice for data analysis and preparation of the manuscript.

MS participated in the design of the experiment, took part in the time course study and gave helpful advice for the preparation of the manuscript.

RAPJr. gave helpful advice for the preparation of the manuscript.

AH designed the study, coordinated the experiments, and helped to draft the manuscript.

## References

[B1] Desqueyroux H, Pujet JC, Prosper M, Le Moullec Y, Momas I (2002). Effects of air pollution on adults with chronic obstructive pulmonary disease. Arch Environ Health.

[B2] Peel JL, Tolbert PE, Klein M, Metzger KB, Flanders WD, Todd K, Mulholland JA, Ryan PB, Frumkin H (2005). Ambient air pollution and respiratory emergency department visits. Epidemiology.

[B3] Stenfors N, Pourazar J, Blomberg A, Krishna MT, Mudway I, Helleday R, Kelly FJ, Frew AJ, Sandstrom T (2002). Effect of ozone on bronchial mucosal inflammation in asthmatic and healthy subjects. Respir Med.

[B4] Savov JD, Whitehead GS, Wang J, Liao G, Usuka J, Peltz G, Foster WM, Schwartz DA (2004). Ozone-induced acute pulmonary injury in inbred mouse strains. Am J Respir Cell Mol Biol.

[B5] McKinney WJ, Jaskot RH, Richards JH, Costa DL, Dreher KL (1998). Cytokine mediation of ozone-induced pulmonary adaptation. Am J Respir Cell Mol Biol.

[B6] Moffatt RK, Hyde DM, Plopper CG, Tyler WS, Putney LF (1987). Ozone-induced adaptive and reactive cellular changes in respiratory bronchioles of bonnet monkeys. Exp Lung Res.

[B7] Wiester MJ, Tepper JS, Winsett DW, Crissman KM, Richards JH, Costa DL (1996). Adaptation to ozone in rats and its association with ascorbic acid in the lung. Fundam Appl Toxicol.

[B8] Harkema JR, Hotchkiss JA, Barr EB, Bennett CB, Gallup M, Lee JK, Basbaum C (1999). Long-lasting effects of chronic ozone exposure on rat nasal epithelium. Am J Respir Cell Mol Biol.

[B9] Crouch EC (2000). Surfactant protein-D and pulmonary host defense. Respir Res.

[B10] Finkelstein JN, Johnston CJ (2004). Enhanced sensitivity of the postnatal lung to environmental insults and oxidant stress. Pediatrics.

[B11] Vandivier RW, Ogden CA, Fadok VA, Hoffmann PR, Brown KK, Botto M, Walport MJ, Fisher JH, Henson PM, Greene KE (2002). Role of surfactant proteins A, D, and C1q in the clearance of apoptotic cells in vivo and in vitro: calreticulin and CD91 as a common collectin receptor complex. J Immunol.

[B12] Gardai SJ, Xiao YQ, Dickinson M, Nick JA, Voelker DR, Greene KE, Henson PM (2003). By binding SIRPalpha or calreticulin/CD91, lung collectins act as dual function surveillance molecules to suppress or enhance inflammation. Cell.

[B13] Atochina EN, Beers MF, Tomer Y, Scanlon ST, Russo SJ, Panettieri RAJ, Haczku A (2003). Attenuated allergic airway hyperresponsiveness in C57BL/6 mice is associated with enhanced surfactant protein (SP)-D production following allergic sensitization. Respir Res.

[B14] Madan T, Reid KB, Singh M, Sarma PU, Kishore U (2005). Susceptibility of mice genetically deficient in the surfactant protein (SP)-A or SP-D gene to pulmonary hypersensitivity induced by antigens and allergens of Aspergillus fumigatus. J Immunol.

[B15] Haczku A, Cao Y, Vass G, Kierstein S, Nath P, Atochina-Vasserman EN, Scanlon ST, Li L, Griswold DE, Chung KF, Poulain FR, Hawgood S, Beers MF, Crouch EC (2006). IL-4 and IL-13 form a negative feedback circuit with surfactant protein-D in the allergic airway response. J Immunol.

[B16] Wong CJ, Akiyama J, Allen L, Hawgood S (1996). Localization and developmental expression of surfactant proteins D and A in the respiratory tract of the mouse. Pediatr Res.

[B17] Fujita M, Shannon JM, Ouchi H, Voelker DR, Nakanishi Y, Mason RJ (2005). Serum surfactant protein D is increased in acute and chronic inflammation in mice. Cytokine.

[B18] Cao Y, Tao JQ, Bates SR, Beers MF, Haczku A (2004). IL-4 induces production of the lung collectin surfactant protein-D. J Allergy Clin Immunol.

[B19] Casey J, Kaplan J, Atochina-Vasserman EN, Gow AJ, Kadire H, Tomer Y, Fisher JH, Hawgood S, Savani RC, Beers MF (2005). Alveolar Surfactant Protein D Content Modulates Bleomycin Induced Lung Injury. Am J Respir Crit Care Med.

[B20] Botas C, Poulain F, Akiyama J, Brown C, Allen L, Goerke J, Clements J, Carlson E, Gillespie AM, Epstein C, Hawgood S (1998). Altered surfactant homeostasis and alveolar type II cell morphology in mice lacking surfactant protein D. Proc Natl Acad Sci U S A.

[B21] Beatty AL, Malloy JL, Wright JR (2005). Pseudomonas aeruginosa degrades pulmonary surfactant and increases conversion in vitro. Am J Respir Cell Mol Biol.

[B22] Grubor B, Gallup JM, Ramirez-Romero R, Bailey TB, Crouch EC, Brogden KA, Ackermann MR (2004). Surfactant protein D expression in normal and pneumonic ovine lung. Vet Immunol Immunopathol.

[B23] Grubor B, Gallup JM, Meyerholz DK, Crouch EC, Evans RB, Brogden KA, Lehmkuhl HD, Ackermann MR (2004). Enhanced surfactant protein and defensin mRNA levels and reduced viral replication during parainfluenza virus type 3 pneumonia in neonatal lambs. Clin Diagn Lab Immunol.

[B24] Soerensen CM, Holmskov U, Aalbaek B, Boye M, Heegaard PM, Nielsen OL (2005). Pulmonary infections in swine induce altered porcine surfactant protein D expression and localization to dendritic cells in bronchial-associated lymphoid tissue. Immunology.

[B25] Barr BC, Hyde DM, Plopper CG, Dungworth DL (1990). A comparison of terminal airway remodeling in chronic daily versus episodic ozone exposure. Toxicol Appl Pharmacol.

[B26] Hudson B, Flemming J, Sun G, Rand TG (2005). Comparison of immunomodulator mRNA and protein expression in the lungs of Stachybotrys chartarum spore-exposed mice. J Toxicol Environ Health A.

[B27] Bachurski CJ, Ross GF, Ikegami M, Kramer BW, Jobe AH (2001). Intra-amniotic endotoxin increases pulmonary surfactant proteins and induces SP-B processing in fetal sheep. Am J Physiol Lung Cell Mol Physiol.

[B28] Li L, Hamilton RFJ, Holian A (2000). Protection against ozone-induced pulmonary inflammation and cell death by endotoxin pretreatment in mice: role of HO-1. Inhal Toxicol.

[B29] Leth-Larsen R, Garred P, Jensenius H, Meschi J, Hartshorn K, Madsen J, Tornoe I, Madsen HO, Sorensen G, Crouch E, Holmskov U (2005). A common polymorphism in the SFTPD gene influences assembly, function, and concentration of surfactant protein D. J Immunol.

[B30] Heidinger K, Konig IR, Bohnert A, Kleinsteiber A, Hilgendorff A, Gortner L, Ziegler A, Chakraborty T, Bein G (2005). Polymorphisms in the human surfactant protein-D (SFTPD) gene: strong evidence that serum levels of surfactant protein-D (SP-D) are genetically influenced. Immunogenetics.

[B31] Brazil TJ, Dagleish MP, McGorum BC, Dixon PM, Haslett C, Chilvers ER (2005). Kinetics of pulmonary neutrophil recruitment and clearance in a natural and spontaneously resolving model of airway inflammation. Clin Exp Allergy.

[B32] Schaub B, Westlake RM, He H, Arestides R, Haley KJ, Campo M, Velasco G, Bellou A, Hawgood S, Poulain FR, Perkins DL, Finn PW (2004). Surfactant protein D deficiency influences allergic immune responses. Clin Exp Allergy.

[B33] Yoshida M, Whitsett JA (2006). Alveolar macrophages and emphysema in surfactant protein-D-deficient mice. Respirology.

[B34] White MR, Crouch E, Vesona J, Tacken PJ, Batenburg JJ, Leth-Larsen R, Holmskov U, Hartshorn KL (2005). Respiratory Innate Immune Proteins Differentially Modulate the Neutrophil Respiratory Burst Response to Influenza A Virus. Am J Physiol Lung Cell Mol Physiol.

[B35] Tacken PJ, Hartshorn KL, White MR, van Kooten C, van de Winkel JG, Reid KB, Batenburg JJ (2004). Effective targeting of pathogens to neutrophils via chimeric surfactant protein D/anti-CD89 protein. J Immunol.

[B36] Liu CF, Chen YL, Shieh CC, Yu CK, Reid KB, Wang JY (2005). Therapeutic effect of surfactant protein D in allergic inflammation of mite-sensitized mice. Clin Exp Allergy.

[B37] He Y, Crouch EC, Rust K, Spaite E, Brody SL (2000). Proximal promoter of the surfactant protein D gene: regulatory roles of AP-1, forkhead box, and GT box binding proteins. J Biol Chem.

[B38] Johnston RA, Schwartzman IN, Flynt L, Shore SA (2005). Role of interleukin-6 in murine airway responses to ozone. Am J Physiol Lung Cell Mol Physiol.

[B39] Ward NS, Waxman AB, Homer RJ, Mantell LL, Einarsson O, Du Y, Elias JA (2000). Interleukin-6-induced protection in hyperoxic acute lung injury. Am J Respir Cell Mol Biol.

[B40] Lim S, Caramori G, Tomita K, Jazrawi E, Oates T, Chung KF, Barnes PJ, Adcock IM (2004). Differential expression of IL-10 receptor by epithelial cells and alveolar macrophages. Allergy.

